# Self-rated health trajectories: A dynamic time warp analysis

**DOI:** 10.1016/j.pmedr.2021.101510

**Published:** 2021-08-10

**Authors:** Brian M. Doornenbal, Renz Bakx

**Affiliations:** a Leiden University Medical Center, the Netherlands; b Salut., the Netherlands

**Keywords:** Self-rated health, Health trajectories, Dynamic time warping, Clustering analysis, Dutch

## Abstract

Self-rated health (SRH), individuals’ overall perception of their health, is a key predictor of health events. To target disease prevention efforts, it is important to understand how SRH develops over time. The goal of this short communication is to find prototypic SRH trajectories by applying dynamic time warping, a time series comparison technique initially developed for speech recognition. Revealing prototypic SRH trajectories can help direct disease prevention efforts towards trajectories that are more likely to result in adverse health events. Based on data from a Dutch representative sample of 2,154 individuals, our dynamic time warp analysis suggests that Dutch individuals do not typically show a steady growth or decline in SRH. Instead, we identified four relatively stable SRH trajectories that differed in average SRH. One of these trajectories is a path of consistent low SRH.

## 1 Introduction

Self-rated health (SRH), individuals’ overall perception of their health, is a key predictor of health events. Individuals who report lower levels of health care, among others, more likely to experience a stroke (Mavaddat et al., 2016), get hospitalized (DeSalvo et al., 2005), and suffer from cardiovascular diseases (Mavaddat et al., 2014). To predict health events, SRH is suggested to be at least as important as more objective health data such as functional limitations and specific medical conditions (Blazer, 2008). This predictive value is the result of the more holistic information contained in SRH measurements, that dynamically blend various health domains related to previous health experience, current health burdens, and future health expectations (Ayyagari et al., 2012).

Although much is known about the predictive value of SRH, less is known about intra-individual changes in SRH (Ayyagari et al., 2012). Given that sharp declines in SRH can predict adverse major health events (Diehr et al., 2001), understanding intra-individual changes in SRH are of vital importance. Many studies show that, on average, SRH slowly declines over time (Bunda and Busseri, 2019, Liang et al., 2005, Sacker et al., 2011). Some studies suggest that SRH changes over time can be categorized into different (non-linear) trajectories (Ayyagari et al., 2012, Liang et al., 2005, Sokol et al., 2017). Knowledge about these trajectories is useful in order to direct disease prevention efforts. Disease prevention efforts could help to intervene with the trajectories associated with major health events.

Previous studies investigating SRH trajectories compared changes in SRH as a function of age (Liang et al., 2005, Sacker et al., 2011, Sokol et al., 2017) and the date of measurement (Ayyagari et al., 2012). This approach helps to understand how SRH changes are related to age and/or events (e.g. a crisis). This approach is less suitable, however, when individuals experience a similar SRH change that moves at a different pace or starts a different moment in time. Some changes in SRH, for example changes induced by life events such as losing/getting a job, are likely to move at a different pace or start at different times. If *actual similar* SRH trajectories move at a different pace or start at a different time, it is probable that the trajectories are *perceived* as *dissimilar*. Thus, while investigating SRH trajectories, it is important to account for local accelerations and decelerations in the time axis.

The goal of this short communication is to find prototypic SRH trajectories by applying dynamic time warping (DTW). This time series comparison technique accounts for local accelerations and decelerations in the time axis (Keogh and Pazzani, 2001, Müller, 2007), making it suitable to compare trajectories that start at different moments in time and/or move at different speeds. DTW was initially developed for speech recognition (Keogh and Pazzani, 2001), but has also proven to be useful in other domains (Müller, 2007), including health research (e.g., Giannoula et al., 2018, Hebbrecht et al., 2020). By revealing prototypic SRH trajectories, the aim of this paper is to help direct preventive medicine efforts towards trajectories that are more likely to result in adverse health events.

## 2 Method

### 2.1 Study sample

In this paper we make use of data of the LISS (Longitudinal Internet studies for the Social Sciences) panel administered by CentERdata (Tilburg University, The Netherlands). The LISS panel is a representative sample of Dutch individuals (Scherpenzeel, 2011). The data from this panel are available for academic research on www.lissdata.nl (see www.lissdata.nl/faq-page#n5512 for information about the ethical approval). In this paper, the sample included 2,154 respondents (49% women) who, with the exception of 2014, rated their health on a yearly basis from 2009 to 2018. The age of the respondents ranged from 16 to 96 (*M* = 56.5, *SD* = 14.1).

## 3 Measurements

To measure SRH, a widely used global measure of self-rated health was employed: Respondents were asked “How would you describe your health, generally?” Responses were rated on a 5-point Likert scale ranging from 1 (=poor health) to 5 (=excellent health). Even though researchers have put forward two interpretations of SRH, as reflecting either a *spontaneous assessment* (i.e., a responsive measurement) of overall health status or an *enduring self-concept* (i.e., a stable measurement) (Bailis et al., 2003), the single-item SRH scale is argued to be as valid, reliable, and sensitive as a multi-item scale for longitudinal research purposes (Macias et al., 2015).

## 4 Analysis

Time series comparison technique DTW was applied to compute the similarity distance between each pair of times series (Keogh and Pazzani, 2001, Müller, 2007). A Sakoe-Chiba Band of 2 was used to match the SRH scores to a maximum of two time point. Based on the similarity distance between the time series, the respondents were clustered into prototypical SRH trajectories. The similarity distance between time series was computed using dtw-python library (Giorgino, 2009). Subsequently, the distance was converted into a (2154, 2154)-proximity matrix, containing the similarity distance in scores between each pair of individuals. Naturally, this matrix was symmetric and contained redundant information. The redundant information was removed using the *SciPy* library’s squareform function (Virtanen et al., 2020). The condensed form of the distance matrix was then used to perform Agglomerative Hierarchical Clustering using the Ward’s minimum-variance method (Murtagh and Legendre, 2014). This clustering works in a “bottom-up” manner in which each SRH trajectory is initially considered as a single-element cluster (i.e. a leaf). Iteratively, the algorithm combines the two clusters that are most similar into a larger cluster (i.e. a node), until all trajectories are combined into one big cluster (i.e. the root). While combining the trajectories into clusters, the Ward’s minimum-variance method chooses to merge clusters that will result in the smallest increase in the value of the sum-of-squares variance.

## 5 Results

**Fig. 1:**
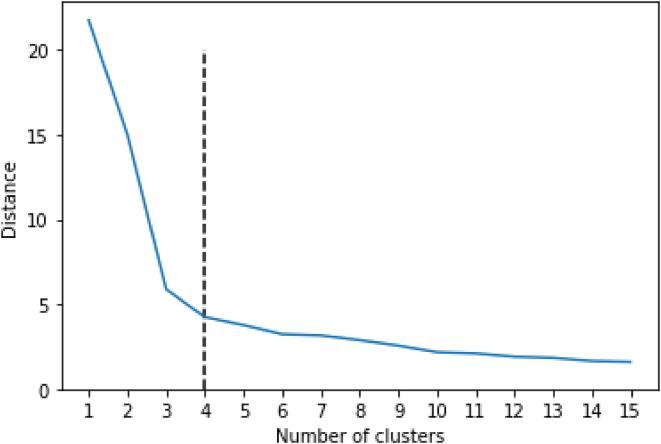
Elbow plot visualizing the average weighted distance against the number of candidate clusters. The chosen number of clusters is shown by the dashed line.

The performance of the clustering algorithm is reported in Fig. 1. In this figure, the distance within the clusters is visualized as a function of the number of candidate clusters. Increasing the number of clusters will naturally decrease the distance within the clusters – the distance will eventually become zero for a number of clusters equal to the number of respondents. As shown in Fig. 1, the distance within the clusters decreases steeply until four candidate clusters are introduced. Following this elbow rule, the data cluster well in four different SRH trajectories.

**Fig. 2:**
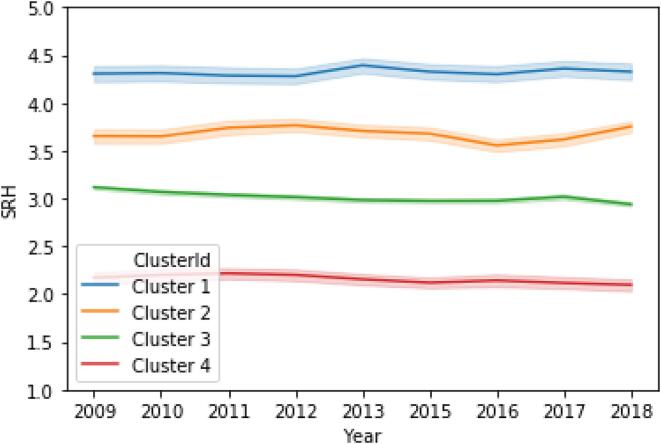
SRH trajectories for four different clusters. *Note.* Self-rated health labels: 1 = poor; 2 = moderate; 3 = good; 4 = very good; 5 = excellent. 95% confidence intervals are depicted by the shaded areas.

Fig. 2 visualizes the four clusters in decreasing order of average SRH. All clusters show relatively flat SRH trajectories. Within each cluster, the respondents display some variability in their SRH, having ups and downs, but seem to stay within a constant bandwidth. The clusters differ from each other in average SRH. Cluster 1 (n = 150; 7.0%) comprises individuals who are consistently positive about their health (*M* = 4.31, *SD* = 0.52), Cluster 2 (n = 308; 14.3%) consists of individuals with a (very) good SRH (*M* = 3.68, *SD* = 0.61), Cluster 3 (n = 1,386; 64.3%) is made up of individuals with a good SRH (*M* = 3.01, *SD* = 0.45), and Cluster 4 (n = 310; 14.4%) consists of individuals scoring consistently low on SRH (*M* = 2.16, *SD* = 0.56). At the within-person level, individuals in Clusters 2 and 4 fluctuated the most in terms of SRH, mostly ranging mostly from good to very good (Cluster 2; 1 SD [3.12, 4.24]) and around moderate SRH (Cluster 4; 1 SD [1.74, 2.58]). Individuals in Clusters 1 and 3 varied within a narrower bandwidth, with SRH ranging mostly from very good to excellent (Cluster 1; 1 SD [3.96, 4.68]) and around good (Cluster 3; 1 SD [2.70, 3.33]).

Table 1 reports the demographics and other personal characteristics for each cluster. We tested the differences among the four clusters on these characteristics by conducting a MANOVA. The MANOVA indicated significant differences on the multivariate combination of the SRH trajectories and personal characteristics: Λ = 0.78, F(15, 5921.81) = 31.24, p < .001, *η^2^* = 0.07. When then conducted ANOVAs to examine the univariate differences in the personal characteristics. These ANOVAs suggested univariate differences in all five personal characteristics (*p* < .05). The effect sizes were largest for medication use (*η^2^* = 0.16). To evaluate differences between the SRH trajectories, we then conducted post hoc pairwise comparisons using Bonferroni correction (α = 0.05). The pairwise comparisons revealed that the individuals in the different SRH trajectories differed especially in age, employment, and medication use. Individuals in clusters that scored higher on SRH often were younger, had a lower Body Mass Index (BMI), and used less often medicine. The proportion of women was significantly (*p* < .05) lower in the “Very Good – Excellent” SRH cluster than in the “Good” SRH cluster. Furthermore, the cluster of individuals from the “Very-good – Excellent” SRH cluster were significantly more often employed than individuals from the clusters “Good” (*p* < .01) and “Moderate” SRH (*p* < .05).

**Table 1:** Demographics and other personal characteristics for each cluster.

Cluster	Gender (woman)	Age	BMI	Employed	Medication use
Very good – Excellent	39% ^c^	50 (16) ^c, d^	24 (3) ^b, c, d^	71% ^c, d^	21% ^b, c, d^
Good – Very good	44%	54 (15) ^c, d^	25 (3) ^a, c, d^	62%	34% ^a, c, d^
Good	52% ^a^	57 (14) ^a, b, d^	26 (4) ^a, b, d^	59% ^a^	56% ^a, b, d^
Moderate	51%	60 (12) ^a, b, c^	28 (5) ^a, b, c^	60% ^a^	89% ^a, b, c^

*F*(3,2150)	4.92	22.80	33.20	3.72	136.78
*η^2^*	0.01	0.03	0.04	0.01	0.16

*Note.* Percentages denote the average status of individuals; Medication use (reverse coded) measured as “I do not take any medicine”; Standard deviations reported in between brackets; Multivariate analysis of variance (MANOVA): Λ = 0.78, *F*(15, 5921.81) = 31.24, *p* < .001, *η^2^* = 0.07; ^a^ Significantly different from mean score “Very Good – Excellent” cluster in Bonferroni-adjusted post hoc tests (*p* < .05); ^b^ Significantly different from mean score “Good – Very Good” cluster in Bonferroni-adjusted post hoc tests (*p* < .05); ^c^ Significantly different from mean score “Good” cluster in Bonferroni-adjusted post hoc tests (*p* < .05); ^d^ Significantly different from mean score “Moderate” cluster in Bonferroni-adjusted post hoc tests (*p* < .05); BMI = Body Mass Index (kg/m^2^).

## 6 Discussion

The aim of this short communication was to find prototypic SRH trajectories by applying DTW. The data used for this study clustered well into four different trajectories. All of these trajectories showed a relatively flat SRH pattern. The most common trajectory (64.3%) was of “good” SRH. A small group of individuals (7.0%) experience a trajectory of “very good” to “excellent” health, whereas the remaining individuals (14.3% and 14.4%) consistently scored their health as either “moderate” or ”very good”.

By contributing to the understanding of SRH trajectories, the ultimate goal of this study was to identify opportunities to prevent adverse health events. Previous research suggests that sharp declines in SRH predicts adverse major health events (Diehr et al., 2001). In the present study, we did not find prototypic SRH trajectories characterized by clear declines in SRH. However, we did find that about 14.4% of the respondents followed a SRH trajectory of consistent moderate health. These individuals often used medicine, on average 89% of the time. Given that lower SRH is associated with adverse health events (Mavaddat et al., 2014, Mavaddat et al., 2016), our findings suggest that disease prevention initiatives should consider focusing on helping individuals break free from a consistent moderate SRH.

In contrast to previous studies (e.g., Ayyagari et al., 2012, Liang et al., 2005, Sokol et al., 2017), we did not find non-linear SRH trajectories. Previous studies found non-linear SRH trajectories related to among others age (Liang et al., 2005, Sacker et al., 2011, Sokol et al., 2017) and (socioeconomic) status (Ayyagari et al., 2012). We studied SRH while allowing for accelerations and decelerations in the time axis. This technique (i.e. DTW) can help to find non-linear health trajectories (e.g., Giannoula et al., 2018, Hebbrecht et al., 2020), but did not suggest non-linearities based on the current data. Possibly, our findings would have been different when we would have focused on a specific group (e.g. Ayyagari et al., 2012), studied shorter term changes (e.g. Hebbrecht et al., 2020), or considered SRH in relation to age (e.g. Sokol et al., 2017). In future research, scholars should also consider different time series techniques, such as derivative dynamic time warping (DDTW). Possibly, individuals across the identified clusters show different non-linear changes (e.g., an improving trajectory, a flat trajectory, and a decreasing trajectory). Time series technique derivative dynamic time warping (DDTW), would be suited to identify such trajectories. DDTW is a technique developed based on DTW and is suitable for filtering out differences on the Y-axis (Keogh and Pazzani, 2001). Instead of considering the Y-values of datapoints, DDTW considers changes in Y-values. If future research points out non-linear trajectories scattered across different levels of SRH, it still should consider the differences on the Y-axis. Given that lower SRH is associated with adverse health events (Mavaddat et al., 2014, Mavaddat et al., 2016), a decreasing trajectory might be more problematic for individuals with a lower SRH. Future research should also consider using Latent Growth Mixture Modelling (LGMM) to estimate parameters that define the different trajectories (e.g., Colder et al., 2001). Such parameters contribute to a better understanding of the stability of the SRH trajectories. This short communication provides an overview of SRH trajectories in The Netherlands. Other scholars have found similar flat (but declining) SRH trajectories in Denmark, Germany, Japan, the UK, and the USA (Liang et al., 2005, Sacker et al., 2011, Sokol et al., 2017). In order to develop disease prevention efforts that improve the overall health, it is crucial to understand how contextual variables relate to the found trajectories. Besides the studied demographic factors, lifestyle, and functional status can affect SRH trajectories (Ayyagari et al., 2012, Sokol et al., 2017). If lifestyle is an important predictor of SRH trajectories indeed, then disease prevention efforts could try to alter SRH trajectories and, ultimately, contribute to the overall health.

## CRediT authorship contribution statement

**Brian M. Doornenbal:** Conceptualization, Methodology, Writing - original draft, Writing - review & editing, Supervision. **Renz Bakx:** Writing - review & editing, Formal analysis.

## Declaration of Competing Interest

The authors declare that they have no known competing financial interests or personal relationships that could have appeared to influence the work reported in this paper.
